# Motivation, Barriers, and Trust: Supporting Early Breast Cancer Treatment: a commentary

**DOI:** 10.1177/17449871251347825

**Published:** 2025-08-28

**Authors:** Katarina Sjövall

**Affiliations:** Senior Lecturer in Nursing, Faculty of Health Sciences, Kristianstad University, Kristianstad, Sweden

Major improvements have been made over recent decades in diagnosis and treatment of breast cancer, and improvements in survival rates have followed. Achieving positive survival rates is based on early diagnosis and early treatment initiation. The paper ‘Determinants of Initiating Cancer Treatment among Breast Cancer Survivors: A Descriptive Qualitative Study’ explored perceived benefits, barriers and challenges related to initiation of cancer treatment among women with breast cancer (Almallah et al., 2025). Through qualitative interviews with breast cancer survivors undergoing cancer treatment, health beliefs and cues to initiate treatment were explored. Participating women described how those positive beliefs, such as personal, social, and cancer-related benefits, motivated them to initiate early treatment. Delaying treatment was perceived as a threat, expressed as existential distress. Perceived barriers to initiating early treatment were related to potential treatment toxicity and lack of information, to practical consequences of treatment in daily living, and financial consequences. Participants also reported feeling confident in their ability to decide and initiate early treatment.

For many women being diagnosed with breast cancer causes various levels of emotional and existential distress (Campbell-Enns and Woodgate, 2015; Gall and Bilodeau, 2020). Although most women do not experience any symptoms from the breast cancer itself, treatment might have a substantial impact on the woman’s quality of life. Several studies report experiences of various challenges related to changed female identity and body image, along with an increased uncertainty about life (e.g. Ahn and Suh, 2023; Paterson et al., 2016). This study illustrates how women’s health beliefs related to treatment might have an impact on the initiation of early treatment, being perceived either as a benefit or a barrier. To support the woman through diagnosis, treatment decision and initiation, personal beliefs and needs should be assessed alongside social determinants. As noted by Almallah et al. (2025), nurses have a primary role in this; however, the whole healthcare team should be involved. This is also supported by the finding in this study that women’s self-confidence in their ability to initiate early treatment was partly based on trust in healthcare professionals, and their knowledge about breast cancer and the treatment.

Some parts of the findings might have limited transferability, given that healthcare systems do differ internationally, for example health insurance coverage and approval as a barrier for treatment. However, financial difficulties related to breast cancer treatment are reported from studies around the world and should therefore be considered in the process of treatment decision and initiation (see Gharzai et al., 2021). In addition, as the authors point out, further research is needed to focus on participants who have delayed treatment as they might contribute to further knowledge and understanding.

Findings from the study explore the understanding of women’s perspective during the process of decision-making around, and initiation of, breast cancer treatment. The study highlights the importance of addressing each woman’s personal beliefs, motivation and barriers to treatment. Nurses can play a vital role in this, by providing a holistic care from the very first day of breast cancer diagnosis and continuously through treatment and rehabilitation.
